# Potential Resistance of SARS-CoV-2 Main Protease (Mpro) against Protease Inhibitors: Lessons Learned from HIV-1 Protease

**DOI:** 10.3390/ijms23073507

**Published:** 2022-03-23

**Authors:** János András Mótyán, Mohamed Mahdi, Gyula Hoffka, József Tőzsér

**Affiliations:** 1 Department of Biochemistry and Molecular Biology, Faculty of Medicine, University of Debrecen, 4032 Debrecen, Hungary; motyan.janos@med.unideb.hu (J.A.M.); mohamed@med.unideb.hu (M.M.); hoffka.gyula@med.unideb.hu (G.H.); 2 Doctoral School of Molecular Cell and Immune Biology, University of Debrecen, 4032 Debrecen, Hungary

**Keywords:** SARS-CoV-2, main protease (Mrpo), HIV-1, resistance, drug resistance, PAXLOVID, PF-07321332, nirmatrelvir, protease inhibitor

## Abstract

Coronavirus disease 2019 (COVID-19), caused by the severe acute respiratory syndrome 2 (SARS-CoV-2), has been one of the most devastating pandemics of recent times. The lack of potent novel antivirals had led to global health crises; however, emergence and approval of potent inhibitors of the viral main protease (Mpro), such as Pfizer’s newly approved nirmatrelvir, offers hope not only in the therapeutic front but also in the context of prophylaxis against the infection. By their nature, RNA viruses including human immunodeficiency virus (HIV) have inherently high mutation rates, and lessons learnt from previous and currently ongoing pandemics have taught us that these viruses can easily escape selection pressure through mutation of vital target amino acid residues in monotherapeutic settings. In this paper, we review nirmatrelvir and its binding to SARS-CoV-2 Mpro and draw a comparison to inhibitors of HIV protease that were rendered obsolete by emergence of resistance mutations, emphasizing potential pitfalls in the design of inhibitors that may be of important relevance to the long-term use of novel inhibitors against SARS-CoV-2.

## 1. Introduction

The pandemic caused by the betacoronavirus severe acute respiratory syndrome 2 (SARS-CoV-2) has caught the world by surprise. As of the date of writing this manuscript, the virus had resulted in over 470 million infections and more than 6 million deaths worldwide, according to the World Health Organization (WHO; https://covid19.who.int/) (accessed date on 20 February 2022). While no novel anti-SARS-CoV-2 drugs are currently being marketed, a great interest had risen in repurposing already available drugs in hope of temporarily halting the spread of the infection.

Of the first antivirals to be repurposed were the human immunodeficiency virus (HIV) protease inhibitors (PIs) lopinavir and ritonavir, which possess an excellent efficacy against the HIV-1; however, clinical experience as well as in vitro studies have later shown that they are not effective against SARS-CoV-2 [1,2,3]. Currently, the only approved and widely used antiviral with agreeable potency against SARS-CoV-2 is the viral RNA-dependent RNA polymerase (RdRp) inhibitor remdesivir, and more recently, a novel protease inhibitor was granted emergency approval by the Food and Drug Administration (FDA), and it is thought to be the most efficacious anti-SARS-CoV-2 drug to date.

Nirmatrelvir (PF-07321332) targets the main protease (Mpro) of SARS-CoV-2, and while the initial trial results and in vitro studies have been very promising, with reportedly an efficacy in the nanomolar concentrations, it is of vital importance to continuously assess and analyze the potency of such drugs targeting proteases of RNA viruses. Given their lack of efficient proof-reading mechanisms and their extraordinary ability to adapt and overcome selective pressures, RNA viruses and coronaviruses, for that matter, are prone to evade antiviral suppression through substitution of target amino acid residues.

As an inherent feature of their replication cycle, RNA viruses, such as coronaviruses and retroviruses, are error prone, hence possessing a relatively large mutation capability. Therefore, in the case of HIV-1, we consider the virus replicating in a patient as a quasispecies that cannot be described by one sequence although there is typically a predominant one. However, whenever an antiretroviral therapy is utilized, this shifts towards a form that is more or less resistant to the drug. The quasispecies idea has also been introduced into coronavirus infections [4,5] due to the error-prone nature of the drug-resistance development and is inevitable in the future whenever antiviral drugs are utilized. What remains to be determined is its degree. Studies on HIV-1 PR drug-resistant mutants provide an excellent example on how enzyme-substrate interactions can be maintained by mutations that cause loss of interactions with the inhibitors. This is independent from the fact that the mechanism of the two enzymes are different. Therefore, our hypothesis is that using the knowledge we gained from HIV PR, we can predict drug-resistant mutations that might occur in SARS-CoV2 PR inhibitor use.

In this review, we explore the binding properties of nirmatrelvir to the Mpro of SARS-CoV-2 and delineate binding target residues in the complex, mutations of which may interfere with the inhibitor’s binding efficacy, learning vital lessons from history by drawing a comparison to HIV PIs, most of which have now been rendered ineffective as a result of emergence of resistance-inducing mutations.

## 2. RNA Viruses

Recent challenging pandemics (AIDS, influenza, COVID-19, Ebola outbreaks) were caused by RNA viruses. As compared to viruses having DNA as the genome, viruses having RNA genomes tend to have higher mutation rates, as they are copied less accurately, even though virus-encoded RNA-dependent RNA polymerases responsible for the replication of these genomes do have some repair mechanisms.

Many viruses require a step called maturation as part of their replication cycle to become infectious. This process generally involves structural changes in the virus particle that may result from specific proteolytic cleavages or conformational changes, which occur in proteins during or after assembly. Virus-encoded proteases are frequently involved in maturation [6] and are usually highly specific for particular amino acid sequences and structures typically for only a few peptide bonds in large and complex virus proteins. Proteases often play an essential function in the replication of a variety of viruses, including those responsible for human diseases. The most well-characterized examples are human immunodeficiency virus type 1 and 2 (HIV-1 and HIV-2), hepatitis viruses, and coronaviruses (CoVs), including severe acute respiratory syndrome coronavirus (SARS-CoV) and severe acute respiratory syndrome coronavirus 2 (SARS-CoV-2, also referred to as 2019-nCoV).

Retroviruses are enveloped viruses containing a non-segmented, single-stranded (+)RNA genome. They can be classified into seven genera, the most prominent of which are the HIV-1 and HIV-2 viruses, which belong to the *Lentivirus* genus of the *Retroviridae* family and are the causative agents of acquired immunodeficiency syndrome (AIDS). A unique feature of lentiviruses is that, in contrast to other retroviruses, they are capable of infecting non-dividing cells. HIVs mainly attack the CD4 T lymphocytes of the immune system [7]. The genomes of HIVs contain the three main genes (*gag*, *pol,* and *env*) as well as accessory genes. The structural proteins of the virus, such as the matrix (MA), capsid (CA), and nucleocapsid (NC), are encoded by the *gag* gene and the *pol* codes for the viral enzymes (reverse transcriptase, RT; integrase, IN; protease, PR), while the *env* encodes the surface glycoprotein (SU) and transmembrane (TM) proteins [8]. The first step of the retroviral replication cycle is the attachment of the virion to the target cell via interacting with CD4 as primary and CCR5 or CXCR4 as co-receptors. Fusion of the cell membrane and the viral envelope enables the entry of the virus, followed by synthesis of the retroviral DNA by reverse transcription using the genomic RNA as a template, which is the most error-prone step of the HIVs life cycle [9]. Thereafter, viral DNA is integrated into the genome of the cell, and the cells with integrated proviruses can permanently produce replication-competent HIV virions [10]. The reverse transcription and the integration are unique for the retroviral life cycle [7]; these steps are missing from the replication cycles of other viruses, including CoVs [11]. Viral RNA molecules are transcribed from the integrated proviral DNA and can be used for the translation of viral polyproteins. Genomic RNA and the viral proteins are assembled into immature virions, which become infectious only after limiting proteolysis of the polyproteins into functional units by the viral protease (maturation) [8,12,13,14].

CoVs are a family of enveloped non-segmented (+)RNA viruses that are distributed widely among mammals and birds and many other wild animals, causing principally respiratory or enteric diseases. They usually infect their hosts in a species-specific manner, and infections can be acute or chronic. Infections are transmitted mainly via respiratory and fecal-oral routes. The outbreaks of SARS in 2002 and the Middle East respiratory syndrome (MERS) in 2012 demonstrated the possibility of animal-to-human and human-to-human transmission of newly emerging CoVs, followed by the outbreak of COVID-19 originally in Wuhan (China), causing a pandemic in early 2020 [15]. This viral subfamily includes four genera: alpha-, beta-, gamma-, and deltacoronavirus. SARS-CoV, the MERS coronavirus (MERS-CoV), and SARS-CoV-2 belong to the *Betacoronavirus* genus [16].

The genome of CoVs is positive-sense, single-stranded RNA that is capped and polyadenylated; these viruses have the largest genomes among all RNA viruses (~30 kb). Unlike most eukaryotic mRNAs, CoVs genomes contain multiple ORFs [17,18]. The genomic RNA is used as template to directly translate polyprotein 1a/1ab (pp1a/pp1ab), which encodes non-structural proteins (nsps). Four structural proteins are essential for virion assembly and infection of CoVs [19]. Homotrimers of spike (S) proteins make up the spikes on the viral surface, and they are responsible for attachment to host receptors. The membrane (M) protein has three transmembrane domains, and it shapes the virions, promotes membrane curvature, and binds to the nucleocapsid (N). The envelope (E) protein plays a role in virus assembly and release, and it is involved in viral pathogenesis. The N protein binds virus RNA genome and is also an antagonist of interferon (IFN) and viral-encoded repressor of RNA interference, which appears to be beneficial for viral replication. After attachment and entry, during the process of uncoating, the genomic RNA becomes available [11,20]. The positive-sense genome, which also serves as the first mRNA of infection, is translated into the enormous replicase polyprotein. The replicase then uses the genome as the template for the synthesis via negative-strand intermediates of both progeny genomes and a set of subgenomic mRNAs. The replication transcription centers (RTCs) are closely associated with double-membrane vesicles (DMVs), which are proposed to be adopted from the modified ER, possibly by the combined activities of nsp3, nsp4, and nsp6. A set of subgenomic RNAs (sgRNAs) are synthesized by RTC. These negative-strand sgRNAs serve as the templates for the production of subgenomic mRNAs (+sgRNAs) [21,22]. The S, E, and M proteins are synthesized and anchored on the endoplasmic reticulum (ER), whereas the N protein is translated in the cytosol. Assembly takes place in the ER-Golgi intermediate compartment (ERGIC), and mature virions are released via smooth-walled vesicles by exocytosis [23]. At the cell surface, S protein can result in the fusion of an infected cell with adjacent, uninfected cells, leading to the formation of large, multinucleate syncytia. This enables the spread of infection independent of the action of extracellular virus, thereby providing some measure of escape from immune surveillance [18,24].

## 3. Proteases Inhibitors and Mutations

### 3.1. HIV-1 Protease and Protease Inhibitors

The virally coded PR of HIV viruses is one of the enzymes required for viral replication. HIV PR belongs to the group of aspartic proteases, named after the conserved catalytic residues Asp-Thr/Ser-Gly at the catalytic site. It is a homodimeric enzyme composed of two identical polypeptide chains, each containing 99 residues. During catalysis, the catalytic triad located at the hydrophobic core of the enzyme mediates proteolytic processing of the substrate through a coordination of a water molecule, leading to the hydrolysis of the target peptide bond. The interaction between the PR and its substrate is governed by a series of structural changes orchestrated by movement of the flap region that is present at the top of the dimer as well as internal movements within the core of PR. Evidence suggests that interaction of at least seven amino acid residues between the substrate and the PR is required for successful proteolysis [25]. It is important to note that the HIV PR binds the substrate in an asymmetric manner, interacting with its peptide backbone in addition to side chains extending into the substrate binding subsites, facilitated by a series of hydrogen bond interactions between C=O and NH groups of the inhibitor and the PR [26].

Given its pivotal role in the viral life cycle, processing polyprotein precursors post-translationally into mature active proteins and hence being indispensable to viral maturation, the PR became one of the first targets for antiretroviral therapies, and the introduction highly active anti-retroviral therapy (HAART) with the availability of first-generation HIV-1 PR inhibitors provided the possibility to re-classify AIDS from deadly disease to a chronic one. However, the quick appearance of drug resistance caused by mutations mainly in the PR gene to diminish PR-inhibitor interactions while maintaining efficient enzyme–substrate interactions initiated a long-term race between drug design and drug resistance, leading to the development of second- and third-generation inhibitors.

Currently, nine PR inhibitors are approved by the FDA, out of which less than a handful remain in production today. The so-called second-generation inhibitors, such as darunavir, lopinavir, and atazanavir, are still being prescribed to date as second-line regimens in combination with reverse transcriptase (RT) inhibitors or inhibitors of the viral integrase (IN). These newer drugs, in addition to third-generation inhibitors in the pipeline, have practically limited the use of first-generation inhibitors, as they were specifically designed to address resistance-inducing mutations selected by the older drugs, improving bioavailability and dosing frequency and minimizing the side effects [27].

Generally speaking, most PIs of HIV are competitive active-site binders, containing a hydroxyethylene core, mimicking the transition state of proteolysis, and preventing cleavage of the inhibitor by the enzyme. They possess functional groups that come into contact with the same residues in the active site of the mature protease dimer, forming a “lock” configuration in addition to being significantly smaller than the PR’s natural substrates. The cleavage sites of HIV-1 PR are diverse, and there is no consensus recognition sequence. In addition, its specificity is very strongly sequence context dependent, and this feature is shared by the retroviral PRs [28]. The particular substrate sequence is not an exclusive determinant of the specificity; rather, the volume (space) filled by the substrate is crucial for efficient binding. The substrate binding pockets of HIV-1 PR overlap, and interactions may occur between the side chains of the substrate [29]. Analysis of crystal structures revealed that the target sequences of the PR have a conserved shape, which was considered to be the basis of the recognition [30]. The space filled by the substrates is referred to as substrate envelope, and accordingly, the space filled by the inhibitor molecules is referred to as inhibitor envelope. Indeed, if we overlay the inhibitors on the substrate envelope, these common binding sites would be identifiable, most commonly between P3 and P2’ sites (based on nomenclature introduced by Schechter and Berger, where the inhibitors tend to interact with specific residues in the viral PR. Therefore, it is easy to comprehend how mutation of these anchoring residues may lead to a multi-drug resistant protease and subsequently treatment failure [31]. This is where in silico studies have immensely aided the development of reliable second-generation inhibitors. The design of HIV-1 PR inhibitors using the substrate envelope model was found to be a promising strategy because the drug molecules that optimally fit to and do not protrude from the envelope may be less susceptible to drug resistance [31,32,33]. Tipranavir, for example, a second-generation non-peptidic PI, was designed to be the most flexible of the lot, conforming to multiple variants of the enzyme with critical drug-resistance mutations and inhibiting dimerization of the enzyme. However, even this agile inhibitor was reduced to limited potential in the face of dual or more mutations at certain critical sites [34].

### 3.2. HIV-1 Protease Inhibitor-Associated Mutations and the Mechanism of Resistance

The development of drug-resistance mutations and selection of resistant variants in the case of HIV PR is mainly due to the lack of proofreading mechanism of the RT, resulting in a high error rate during replication [12,35]. The error rate of replication is approximately 1 × 10^−4^; therefore, considering the 10 kbp size of the genome as an average, each replication cycle could introduce a mutation, providing a vast mutation capability of the virus. This appears in drug-naïve patients [36,37,38], wherein transmission of resistance mutations—including those of the HIV PR—may decrease the efficacy of the antiviral therapy. The overall prevalence of transmitted resistance mutations in newly diagnosed and drug-naïve individuals may be high and varies in different countries [39,40]. Therefore, testing for the transmitted drug-resistance-associated mutations prior to commencing anti-retroviral therapy (ART) is important, according to international guidelines [41]. The resistance mutations may also be acquired as a response to selective pressures introduced by antiretroviral drugs. This is further complicated by the wide genetic diversity of HIV since in addition to having two main types (HIV-1 and HIV-2), each type can be classified into 5–8 main groups, respectively, and combination of viral genomes of different subtypes results in circulating recombinant forms (CRFs) lineages derived from inter-subtype recombination. It is therefore not uncommon to see a genetic distances of 25 and 35% across different subtypes [42]. Resistance mutations against HIV PIs were initially detected in patients undergoing monotherapy, and it is important to note that the development of mutations in the context of combination ART is much harder to approach and further complicated by the selective pressure induced by each agent and the cost of resistance to the virus [43].

Initially, first-generation PIs were considered very potent antivirals; however, it soon became apparent that polymorphism in the HIV-1 PR sequence with substitutions at more than 20 amino acid in the sequence rendered those inhibitors of limited functionality [44]. Moreover, there is more than 50% polymorphism in the amino acid sequences between HIV-1 and HIV-2 PRs, which was found to alter the specificity of the protease for certain peptide inhibitors, resulting in reduced susceptibility to the drugs [45,46].

Mutations conferring resistance to the PIs may arise from amino acid substitutions in either the active site or at sites in close proximity to this region [47]. These mutations are commonly referred to as primary ones, which result in the alteration of the substrate binding site and thus interfere with the binding of the inhibitor either directly or indirectly. Secondary mutations, on the other hand, are additional mutations that tend to accumulate under selection pressure and may either intensify or alleviate effects of the primary mutations. It is not uncommon to encounter 1–6 amino acid insertions at various sites in the viral protease sequence during the course of PI-based therapy, which tend to alleviate selection pressure and improve viral replication [48,49].

Of the most commonly encountered PI-associated resistance mutations are those located within the active cavity or the flap region of the PR, forming hydrophobic interactions with the neighboring flap and loop residues. L90M substitution as a primary example is selected by saquinavir, nelfinavir, indinavir, and lopinavir and results in reduced susceptibility to other PIs with the exception of tipranavir and darunavir. This substitution occurs in the hydrophobic core of the protease, near the catalytic aspartate residues. Another example of a multi-drug resistance-inducing mutation is the I54M substitution, a non-polymorphic mutation that was found to be selected primarily by fosamprenavir, lopinavir, and darunavir, resulting in cross-resistance to nelfinavir, indinavir, and saquinavir [50]. Moreover, mutations inducing cross-resistance were also attributed to substitutions at other critical sites, such as positions 10, 36, 46, 48, 71, 77, 82, and 84 [51]. A thorough discussion regarding mutations of HIV-1 PR and their implication in reducing the susceptibility to PI-based regimens was published previously [52,53,54].

Although the mutation frequency in CoVs is less pronounced, already a large number of sequence variations were detected in the absence of any drug-induced selective pressure, implying that in the long-term, we have to face the inevitable appearance of drug-resistance mutations following mass administration of antivirals.

### 3.3. SARS-CoV-2 Mpro

**Figure 1:**
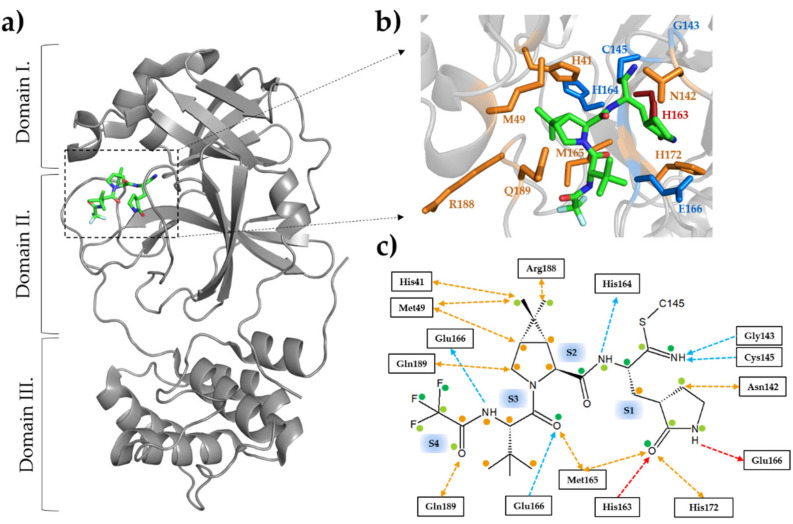
Structure of SARS-CoV-2 Mpro with nirmatrelvir. (**a**) The domain organization is represented based on the crystal structure of the enzyme complexed with nirmatrelvir (7RFS.pdb). The inhibitor is represented as sticks, the carbon atoms are green, the oxygen atoms are red, and the nitrogen atoms are blue. (**b**) Enlarged view of the active site showing the most relevant enzyme-inhibitor interactions. The active-site residues forming at least one main-chain- and side-chain-mediated hydrogen bond are colored by blue and red, respectively. The residues forming apolar interactions are shown in orange. (**c**) Most relevant inhibitor-binding interactions at the active site. The backbone- and side-chain-mediated hydrogen bonds are colored by blue and red, respectively. The direction of the arrow indicates the donated hydrogen atom. Apolar interactions are shown in orange. Colored dots indicate the average of non-bonded contacts for individual atoms. Orange represents 1 contact, light green represent 2–4 contacts, and dark green represent ≥ 5 contacts. The interactions were mapped based on five crystal structures (7vh8, 7si9, 7te0, 7rfs, and 7rfw) using PDBSum [62] and LigPlot+ [63]. See the details in Table 1. An interaction was considered to be relevant if it was present in at least three of the five studied structures.

The SARS-CoV-2 Mpro is also referred to as 3-chymotrypsin-like protease (3CLpro). It functions as a functional homodimer whose structural characteristics highly resemble those of SARS-CoV Mpro [55,56,57]. The full-length protease contains three domains: domain I (10–99 residues) and II (100–184 residues) contain mainly antiparallel β-barrels, while domain III (201–303) has a helical arrangement (Figure 1a). The substrate binding site is located in the cleft between domain I and II, and the long flexible loop that connects domain II and III borders the active site and contains residues that are involved in ligand binding [58]. The catalytic site comprises the His41 and Cys145 residues, which form the catalytic dyad [55,56,57,59].

SARS-CoV-2 Mpro is highly specific and shows strong preference for Leu at P2, Gln at P1, and Gly/Ala/Ser residues at P1’ position [60], but histidine may also occupy P1 position in a small minority of the cleavage sites [61]. The substrate binding sites show high conservation among coronavirus PRs. The S4 is a shallow hydrophobic site, the S3 can bind a wide range of residues due to the exposition of P3 side chain to the solvent, and the S2 and S1 are deep sites. The S1’ site is relatively shallow and preferably binds Ser/Ala residues [57,61].

### 3.4. Inhibitors of SARS-CoV-2 Mpro as (Potential) Therapeutic Drugs

Two years now into the pandemic, clinicians are largely reliant on repurposed broad-spectrum antivirals, such as the RdRp inhibitor remdesevir, to combat mild-to-moderate COVID-19, in addition to monoclonal antibodies and symptomatic therapy. Recently, a novel CoV antiviral targeting the SARS-CoV-2 Mpro has emerged with a very promising potential.

To date, thousands of candidate SARS-CoV-2 Mpro inhibitor compounds have been screened so far in order to identify potent drug candidates [64], but many of them were shown to have no or only limited clinical potential since high concentration was usually required to achieve significant inhibition. Actually, most of the investigational 3CLpro inhibitors are of similar peptidomimetic scaffolds [65].

PF-00835231 is the active metabolite of the PF-07304814, a ketone-based, covalent cysteine protease inhibitor formulated for IV use that contains a phosphonate group to enhance solubility. While it is hard to imagine, PF-00835231 was developed in 2003, organically intended to inhibit the Mpro of SARS-CoV; however, the inhibitor never made it to commercial market given the prompt control of the previous pandemic by public health measures. PF-00835231 was shown to exhibit potent activity against alpha-, beta-, and gammacoronavirus 3CL PRs, with K*i* values ranging from 30 pM to 4 nM. The inhibitor was also found not to be active against a panel of human proteases, with little inhibition of human cathepsin B. Following the outbreak of COVID-19, interest in PF-00835231 was revived, as it was immediately identified as a potential SARS-CoV-2 Mpro inhibitor [66,67,68].

Nirmatrelvir (PF-07321332) is a promising attempt at addressing the early stage of COVID-19 and the concept of prophylaxis. It is a second-generation, reversible covalent inhibitor of SARS-CoV-2 Mpro, binding to the catalytic cysteine residue (C145) via its nitrile warhead [69,70] (Figure 1b,c).

This inhibitor is based on rupintrivir, a peptidomimetic rhinovirus 3C protease inhibitor in development for use against human rhinovirus (HRV) infections that showed good efficacy against all 48 HRV serotypes [71]. Rupintrivir and nirmatrelvir contain the P1 γ-lactam group mimicking glutamine since both HRV and CoV PRs process their substrate after glutamine residues although nirmatrelvir has been better modified to interact with and covalently bind to the active site of SARS-CoV-2 Mpro.

In order to further improve the in vivo stability of nirmatrelvir, the oral SARS-CoV-2 antiviral PAXLOVID (Pfizer; New York, NY, USA) contains the nirmatrelvir combined with HIV-1 PI ritonavir as booster. Ritonavir is not a potent inhibitor of SARS-CoV-2 Mpro [1], but it is applied as a pharmacokinetic enhancer of several other PIs given its potent inhibition of CYP3A4 [72]. Therefore, ritonavir can inhibit the CYP3A4-mediated metabolism of nirmatrelvir and boost its therapeutic concentration.

Nirmatrelvir was found to inhibit the prototypic SARS-CoV-2 Mpro at nanomolar concentration (K*i* = 3.1 nM) [70]. The drug entered phase III clinical trials (NCT04960202, NCT05011513, and NCT05047601). The aim of the active EPIC-HR clinical trial (NCT04960202) is to evaluate the safety of nirmatrelvir/ritonavir among non-hospitalized COVID-19 adult patients (https://clinicaltrials.gov, accessed on 20 February 2022). This NCT04960202 clinical trial is a phase II/III randomized, double blind, placebo-controlled study, and PAXLOVID was shown to be effective in the treatment of mild-to-moderate COVID-19, reducing hospitalization risk by 89% [73]. Additionally, an intravenous candidate (PF-07304814) is also under investigation. This drug contains a phosphonate group to enhance solubility; however, it is yet to gain FDA authorization.

The European Medicines Agency (EMA) has also recommended granting a conditional marketing authorization for PAXLOVID oral antiviral on 27 January 2022; thus, it became the first oral antiviral drug in the European Union as well. PAXLOVID is intended for oral administration and is given twice daily as three pills (two of nirmatrelvir and one of ritonavir) for a total duration of five days.

Another promising peptidomimetic compound in the pipelines is the GC-376, which is an investigational veterinary drug, a broad-spectrum 3CL protease inhibitor. This inhibitor was found to inhibit PRs of CoVs in addition to those of picornaviruses and noroviruses as well [74]. The anti-SARS-CoV-2 activity of this inhibitor was EC_50_ = 3.37 µM while inhibiting the Mpro at a much lesser concentration (IC_50_ value of 0.03 µM) [75].

Finally, boceprevir is an oral ketoamide reversible inhibitor of hepatitis C virus (HCV) NS3 PR. This inhibitor was FDA approved in 2011 and showed excellent efficacy and high selectivity against HCV PR while showing no cross-reactivity with other serine proteases [76]. In the context of SARS-CoV-2, it was found to inhibit Mpro with IC_50_ of 4.13 µM, with an EC_50_ of 1.90 µM against whole-virus infection [75].

### 3.5. Binding of Nirmatrelvir to SARS-CoV-2 Mpro

The enzyme-inhibitor interactions between nirmatrelvir and SARS-CoV-2 Mpro are well established based on molecular dynamical analyses [77,78] and crystallographic studies as well. In February 2022, in total, six structural coordinates of the Mpro complexed with nirmatrelvir became available in the RCSB database; the first was deposited on September of 2021 (7VH8.pdb) [79]. Other crystal structures of the same complex have also been released (Table 1), and a coordinate file of only a single variant is available in the RCSB PDB database so far (7TLL.pdb); this variant contains a P132H mutation [80]. Each structure was determined using X-ray crystallography with ≤2 Å resolution.

In order to combat the resistance mutations of HIV-1 PR, a main strategy of the structure-based inhibitor design is the maximization of the enzyme-inhibitor interactions at the active site by targeting the protein backbone. Based on this concept, the interactions at the active site—mediated by the backbone atoms of the enzyme—are expected to be retained in the case of substitution of the side chains; thus, the mutant enzymes can hardly evade the inhibitors through resistance mutations [81]. Nirmatrelvir bound to the enzyme also forms extensive hydrophobic interactions with the active-site residues; hydrogen bonds are formed mainly at S1 site, where the inhibitor’s nitrile warhead interacts with the catalytic cysteine residue (Cys145). We have summarized the key residues that are responsible for ligand binding based on structures of the enzyme-inhibitor complex (Figure 1c) (Table 1).

The catalytic C145 is able to form a reversible covalent bond via the electrophile nitrile group of nirmatrelvir. The residues that are able to form hydrogen bonds with their backbone atoms include G143, C145, H164, and E166. The G143 and C145 residues interact with the imine nitrogen of the thioimidate inhibitor moiety at the S1 site. The main chain carbonyl oxygen of H164 is able to form a hydrogen bond with an amide nitrogen of the inhibitor. Another hydrogen bond is present between the oxygen atom of the lactam ring and H163. This interaction occurs through the side chain of H163 and can be prone to mutations. Two backbone hydrogen bonds are formed by E166, and these interact with the amide nitrogen prior to the trifluoroacetyl group and the carbonyl oxygen at the S3 site. Besides, E166 form a side-chain hydrogen bond with the amide nitrogen of the lactam ring, which could also be affected by mutations.

**Figure 2:**
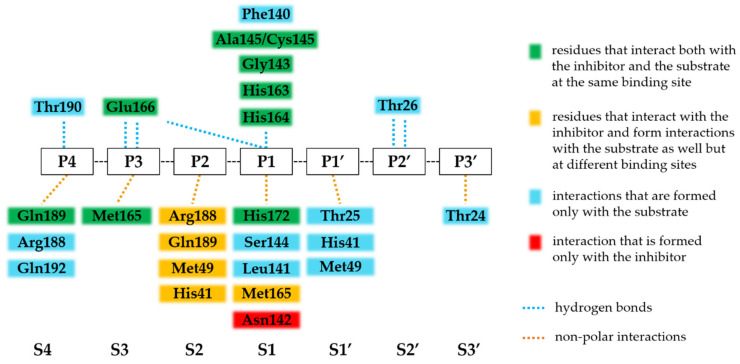
Comparison of substrate- and nirmatrelvir-binding interactions of SARS-CoV-2 Mpro. The interactions are represented based on Table 1. An interaction was considered as relevant if it was present in >50% of the studied enzyme-substrate or enzyme-nirmatrelvir complexes. The enzyme-substrate complexes contain modified catalytic residue (C145A). The substrate binding sites at the active site (S4-S3’) and the substrate residues (P4-P3’) that bind to these sites are labeled based on nomenclature introduced by Schechter and Berger [85], and the scissible bond is located between P1 and P1’ residues.

Additionally, the residues that are involved in the binding of the substrate and the inhibitor are almost identical (Figure 2). Most of the hydrogen bonds that bind the substrate are also formed with the nirmatrelvir. Most H-bonds that are formed with nirmatrelvir at S4-S2 sites are mediated by the main-chain atoms of the active-site residues (Table 1), indicating that the effects of resistance mutations on the backbone-mediated enzyme-inhibitor interactions may be more moderate as compared to the side-chain-mediated contacts. Most of the hydrogen bond interactions are formed at the S1 site. The apolar interactions show a significantly different network for the inhibitor, as it is able to form several interactions via the dimethylcyclopropylproline group at the S2 site. Residues that interact with the inhibitor at S2 site also bind the substrate; however, they also form interactions at different sites. For example, the H41 and M49 residues interact with the P1’ residue of the substrate but also interact with the inhibitor at the S2 site as well. Interestingly, Asn142 is involved in apolar interactions with the inhibitor only; it forms no relevant interactions with the substrate.

**Table 1:** Substrate and inhibitor-binding interactions at the active site of SARS-CoV-2 Mpro. The enzyme-ligand interactions were mapped using LigPlot+ v2.2.4 [63] and based on data available in PDBsum database [62]. H-bonds (H) and non-polar interactions (NP) are also shown. The interactions that are mediated by the side chains of enzyme residues are bold, and asterisk indicates modified catalytic residue (C145A) in the enzyme-substrate complexes. The graphical representation of the interactions is shown in Figure 2.

PDB	7MGR		7MGS		7N89		7VH8		7RFW		7RFS		7SI9		7TE0		7TLL	
Reference	[82]		[82]		[83]		[79]		[70]		[70]		[84]		Unpublished Study		[80]	
Ligand	AVKLQ*NNE		SAVLQ*SGF		SAVLQ*SGF		nirmatrelvir		nirmatrelvir		nirmatrelvir		nirmatrelvir		nirmatrelvir		nirmatrelvir	
Interaction	H	NP	H	NP	H	NP	H	NP	H	NP	H	NP	H	NP	H	NP	H	NP
S5 site		P168		P168 Q189		P168												
S4 site	T190	M165 L167 R188 Q189 Q192	T190	R188 Q189 Q192	T190	R188 Q192		Q189		M165 Q189 T190		M165 Q189 T190		Q189	**Q192**	Q189		M165 Q189 Y190
S3 site	E166 **N142**	M165 Q189	E166	M165	E166	M165	E166	M165	E166	M165	E166	M165	E166	M165	E166	M165	E166	M165
S2 site		H41 M165 Q189		H41 M49 H164 M165	**Q189**	H41 H164 M165 D187		H41 M49 Y54 D187 R188 Q189		H41 M49 Y54 H164 M165 D187 R188 Q189		H41 M49 H164 R188 Q189		H41 M49 Q189		H41 M49 M165 Q189		H41 M49 Y54 D187 R188 Q189
S1 site	F140 G143 A145* H163 H164 **E166**	L141 S144 M165 H172	F140 G143 A145 * H163 H164	L141 H172	F140 G143 A145 * H163 H164 **E166**	L141 N142 S144 H172	F140 G143 **C145** **H163** H164 **E166**	H41 M49 Y54 L141 N142 H172 D187	G143 **C145** **H163**	H41 M49 Y54 F140 N142 H164 M165 H172 D187 T190	**C145** **H163**	H41 M49 F140 L141 N142 G143 H164 M165 H172 T190	F140 **H163** H164 **E166**	H41 M49 N142 M165 H172	**G143** **C145** **H163** H164 **E166**	F140 N142 G143 H172	**C145** **H163** H164 **E166**	H41 F140 L141 N142 G143 S144 H172
S1’ site	G143	T25 H41 M49	**N142**	T25 H41 M49		T25 H41 M49 N142												
S2’ site	T26	T25	T26	T25	T26	T25												
S3’ site		T24		T24		T24 S46												

Based on analysis of the crystal structures of enzyme-substrate complexes, the substrate envelope of SARS-CoV-2 Mpro was also defined [86]. Nirmatrelvir—binding to the S4-S1 sites of the enzyme—was found to fit the substrate envelope, and the sites where the inhibitor protrudes the envelope and which may be most vulnerable to resistance were also mapped. The enzyme-inhibitor interactions were found to be even beyond the substrate envelope (formed with, e.g., M49, N142, E166, and Q189 residues) [86]. Some of these positions are more variable (e.g., M49) and thus may be correlated to resistance development, while E166 is conserved due to its importance in the dimerization of the Mpro [87]. As in the case of HIV-1 PIs, the substrate envelope hypothesis may be a promising strategy of drug design [65] in order to avoid resistance against SARS-CoV-2 Mpro inhibitors.

### 3.6. SARS-CoV-2 Mpro Variants

The SARS-CoV-2 genome sequences are available through databases, such as the Global Initiative on Sharing All Influenza Data (GISAID) [88] and NCBI databases, and additional databases are also available that can aid variant analysis, including COVID-3D online resource [89], SCoV2-MD [90], and SARS-CoV-2 3D [91]. For this review, we obtained data about SARS-CoV-2 variants from the COVID-3D online resource (http://biosig.unimelb.edu.au/covid3d/, accessed on 20 February 2022) [89]. Currently, data are available only for the circulating natural variants of SARS-CoV-2 Mpro, and PI-resistant variants have not been identified to date although multiple studies were conducted for the identification of those residues of SARS-CoV and SARS-CoV-2 PRs that may confer to drug resistance.

**Figure 3:**
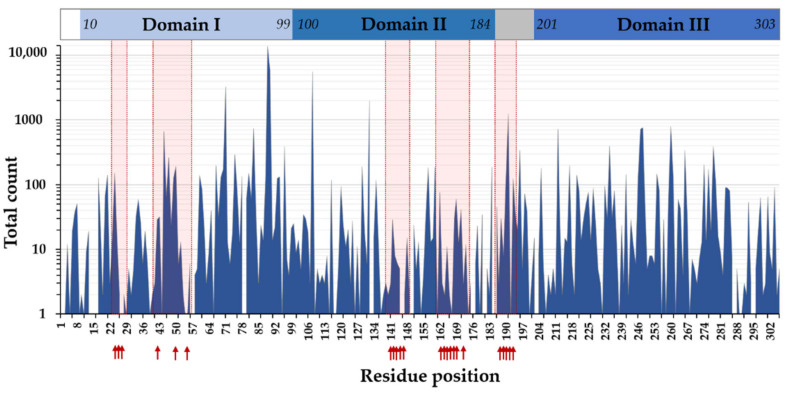
SARS-CoV-2 Mpro sequence variations based on COVID-3D database. The schematic representation of domain organization is also represented, and the arrows show ligand-binding active-site residues based on Table 1.

**Figure 4:**
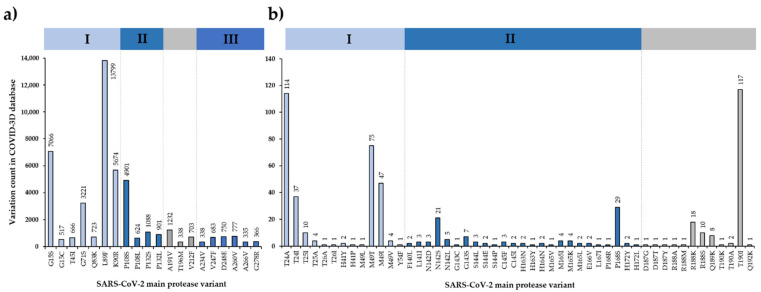
SARS-CoV-2 Mpro sequence variations based on COVID-3D database. Top20 variations: residues with more than 300 known variants (**a**). Mutation frequency of active-site residues involved in the binding of nirmatrelvir (**b**). The database was accessed at 14 January 2022. The bars are colored based on domain organization (domain I–III are blue, while the linker between domain II and III is gray).

The COVID-3D resource includes more than 11,000 variants, which were detected in circulating SARS-CoV-2 genomic sequences [89]. Based on data from COVID-3D database, 291/306 residues of Mpro are known to have at least one variant (Figure 3), and the variable positions, even with a single count of a known variant, cover almost the entire sequence of SARS-CoV-2 Mpro [92]. The most frequently mutated positions obtained from the COVID-3D database (Figure 4) were found to be in good agreement with the missense mutations that are available in the GISAID database [92].

**Figure 5:**
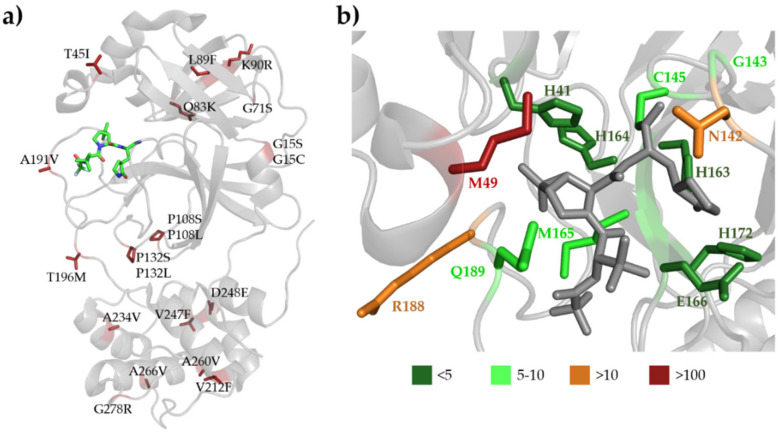
SARS-CoV-2 Mpro sequence variations at the active site. (**a**) The 20 most frequently occurring mutations (based on Figure 3) are shown by red sticks in the crystal structure of the enzyme (7RFS.pdb); the nirmatrelvir bound to the active site is also shown by sticks. (**b**) Enlarged view of the active site showing the inhibitor (gray) and the residues involved in nirmatrelvir binding. The residues are colored according to the number of the known variations in the given position.

The residues that are the most polymorphic in the circulating variants (Figure 4a and Figure 5a) are not directly involved in the binding of nirmatrelvir. Out of the inhibitor-binding residues, the variants of M49 residue have the highest frequency, and the M49T and M49I variants are the most frequent ones. The N142 is also prone to mutations (in total, 29 of the known variants: N142S, N142L, and N142D) (Figure 4b and Figure 5b). Both M49 and N142 residues were found to be located at highly mobile sites and can adopt various conformations; hence, both residues were considered to be potential sites of resistance [86,93].

Different variants of G15 (G15S, G15C), P108 (P108S, P108L), and P132 (P132S, P132L) residues are among the most frequent mutations, indicating higher variability of these residues (Figure 4a). The Y54, N142, T190, A191 [94], H163, and E166 residues at the active site [95] were predicted to be potentially linked to drug resistance. Of these residues, only the A191 is one of the top 20 most frequent variants (Figure 3); the T24, M49, N142, P168, R188, and T190 active-site residues have considerably high variability (Figure 4b). Although A191 residue is not directly involved in inhibitor binding, it is located near the active-site cavity (S4 site). Its mutation may potentially affect the flexibility of the loop that connects domain II and III and contains inhibitor-binding residues (e.g., T190, Q192) [93]. Comparison of ligand-free and -bound structures revealed that the ligand binding-induced conformational changes of the active site play a role in the ligand binding [96]; therefore, the altered conformational flexibilities of the different variants may be correlated to development of resistance.

It was proposed that the cold spots of the active site (or the surface), where the residues are highly conserved and show low mutation frequency, may be promising target for the design of mutation-resistance inhibitors [92]. Mutation-induced structural changes may correlate with the activities of the protease variants [97]; therefore, the circulating mutations that are the most prevalent and/or are potentially related to drug resistance must be investigated, followed by correlation of the structural features with the in vitro enzymatic characteristics.

### 3.7. Efficacy of Nirmatrelvir against SARS-CoV-2 Variants

Data exist regarding the efficacy of nirmatrelvir against SARS-CoV-2 variants and Mpro mutants, and some of the most frequently occurring variants have already been studied by investigating enzymes containing single-point mutations or virus variants (e.g., omicron).

It was found that G15S, T21I, L89F, K90R, and L205V circulating (but not treatment-induced resistance) mutations of SARS-CoV-2 Mpro show similar catalytic features in vitro to the wild-type (wild-type: k_cat_/K_M_ = 0.016 s^−1^µM^−1^, mutants: k_cat_/K_M_ = 0.009–0.015 s^−1^µM^−1^). The inhibitory potential of nirmatrelvir against these variants was also highly comparable that the point mutations did not decrease the efficacy of the inhibitor [98].

A purified SARS-CoV-2 Mpro harboring P132H mutation was studied in vitro [80]. The P132 is one of the most frequently mutated residues of the Mpro (Figure 4a), and the P132H mutation has much lower frequency than P132S and P132L variants. The wild-type and the P132H mutant enzymes exhibited highly comparable catalytic properties: the k_cat_, K_M,_ and k_cat_/K_M_ values were almost identical (wild-type: k_cat_/K_M_ = 22,411 s^−1^M^−1^, P132H: k_cat_/K_M_ = 22,691 s^−1^M^−1^). Nirmatrelvir showed a slightly higher inhibitory potential against the P132H mutant as compared to the wild-type enzyme (wild-type: K*i* = 0.933 nM, P132H: K*i* = 0.635 nM). The highly similar catalytic properties of the wild-type and P132H mutants and their comparable sensitivities towards nirmatrelvir are in agreement with the fact that the P132H mutation induces no remarkable conformational changes and that the active site remains unchanged [80]. Enzyme-inhibitor interactions at the active site of P132H mutant are also identical with those of the wild-type protease (Table 1) since the P132 residue is not located in close proximity to the inhibitor-binding pocket (Figure 5a).

In agreement with results of an in vitro assay, which proved that the purified variants maintained their activity and sensitivity towards nirmatrelvir [98], investigation of infected Calu-3 cells also proved that nirmatrelvir is able to inhibit the prototypic and the omicron SARS-CoV-2 variants as well [99]. Both the level of intracellular viral genomic RNA and the titer of the infectious virus was efficiently decreased by treatment with the protease inhibitor, and the effects were highly comparable in the different strains. Interestingly, the omicron variant showed a slightly higher sensitivity towards the inhibitor in Calu-3 cell model system while not showing higher sensitivity in a human airway organoid model consisting of multiple cell lineages as compared to the wild-type [99]. Additional studies also revealed that different virus variants have similar sensitivity towards nirmatrelvir in Vero E6 cells [100,101]. Despite promising evidence of nirmatrelvir efficacy against different strains [99,100,101], the sequence characteristics of the studied variants have not been revealed, and no information were obtained for the mutation profile of the Mpro. Therefore, a more detailed analysis is required in order to carry out detailed correlation between the inhibitor’s potency and sequence variations of the Mpro and other regions, such as autoproteolytic cleavage sites.

### 3.8. SARS-CoV-2 Cleavage-Site Variants

In addition to ligand-binding site residues, non-active-site mutations of HIV-1 PR are also known to confer resistance against PIs [32,52,102]. Major and minor resistance mutations can be differentiated, with major mutations present at the active site, while the minor mutations may be distant from it. Due to the direct effect on ligand binding, major mutations are more inhibitor specific and can reduce the catalytic efficiency of the protease, decreasing the viral fitness. Minor mutations are considered as accessory mutations, which compensate the effects of major mutations. In addition, mutations that contribute to resistance may be located at the cleavage sites of the protease as well in the Gag and pol polyproteins [103,104,105,106].

Sequences of the circulating SARS-CoV-2 variants also show differences in the autoproteolytic cleavage sites of the viral polyprotein. Mutations are known for all cleavage sites of the Mpro, and some of them show remarkably high frequency (e.g., nsp12-13 site: A598S) (Table 2). Interestingly, the highly conserved P1-Gln residue may be changed to His in multiple cleavage sites (Table 2). Experimental studies have already revealed that minority of the SARS-CoV-2 Mpro cleavage sites contain His residue in P1 position [61] [107]. Specificity profiling studies also showed that SARS-CoV Mpro can cleave substrates containing His in P1 position, and both higher [108] and lower cleavage efficiency [109,110] were observed for the P1-His substrate as compared to that containing Gln in P1 position, respectively.

Cleavage-site mutants were found to emerge in HIV-1-infected patients receiving antiviral therapy due to the positive selection of PI-naïve variants. The growth advantage conferred by the cleavage site mutations is caused by elevated proteolytic activity, as the mutant sites can be cleaved more efficiently by the mutant HIV-1 protease [111,112]. By this mechanism, the HIV-1 cleavage-site mutations can compensate for the effects of major resistance mutations of the protease. Therefore, it remains to be determined how alterations of SARS-CoV-2 cleavage-site sequences change the cleavage efficiencies of the wild-type and mutant PRs and which cleavage-site mutations may be compensatory.

## 4. Conclusions and Perspectives

The COVID-19 pandemic represents a global health crisis. The lack of novel effective antivirals so far had led to an uncontrollable spread of the infection and a sharp rise in mortality rates worldwide. By their nature, RNA viruses have inherently high mutation rates that add to their enhanced virulence, adaptation, and transmission fitness. The multiple variants observed for the prototypical Wuhan SARS-CoV-2 within such a relatively short time is an attestation to their evolvability. However, novel formulations of SARS-CoV-2-specific drugs targeting the viral Mpro may indeed curb the pandemic, at least in the short term.

PAXLOVID, a combination of nirmatrelvir and the booster PI ritonavir, is currently being prepared for mass distribution and has shown an excellent efficacy against SARS-CoV-2 both in in vitro experiments and clinical trials as well; however, we should always be cautious of the perspective of applying mono-therapeutic agents in the context of rapidly evolving RNA virus infection, as history had taught us from previous pandemics that these viruses are capable within a short time of substituting target amino acid residues under treatment pressure in order to evade suppression, exemplified in this review by comparing it to HIV PR and the lessons we have learnt from this decades-old pandemic.

Similar to HIV-1 PR [113], it is not unreasonable to expect resistant mutations arising in SARS-CoV-2 Mpro (and in its cleavage sites), driven by selective drug pressure. The high mutational frequencies of the active-site and dimer-interface hotspots of SARS-CoV-2 Mpro imply potential development of the resistance against inhibitors. The resistant mutations of HIV-1 PR are well characterized, while the occurrence of treatment-induced mutations can be investigated by follow-up studies after the use of PAXLOVID. Thereafter, primary and secondary resistance mutations of the protease may also be distinguished, and the most frequently co-occurring cleavage-site mutations may also be identified. Sufficient structural information are available for the SARS-CoV-2 Mpro as well as HIV-1 PR, which may aid the structure-based drug design. Despite the comparable efficacy of nirmatrelvir against the wild-type protease and its point mutants or variants in different virus strains, currently, we only have information about the PI-naïve variations [80,98,99,100,101]. The currently circulating mutations of the residues that are involved in nirmatrelvir binding are less frequent, and the drug-selective pressure may drive the selection of resistance mutations.

The efficiency of the antiviral therapies are improved in the case of HIV-1 by the application of the combination therapy. In combination ART, a variety of antiretroviral drugs are applied, each targeting different steps of the viral replication cycle, such as reverse transcription (nucleoside and non-nucleoside reverse transcription inhibitors, NRTIs and NNRTIs, respectively), maturation (PIs), entry (entry or fusion inhibitors), and integration (integrase inhibitors, INSTIs) [114]. The simultaneous use of the antivirals which target different viral enzymes can reduce the likelihood of drug resistance development. Due to the ease of resistance development in monotherapy, the co-administration of ritonavir with nirmatrelvir may increase the risk of the development of resistance against HIV PIs in the case of non-diagnosed HIV-1 infections [115]. Therefore, in the case of SARS-CoV-2, combination therapies may decrease the risk of resistance development against the protease inhibitor. The therapeutic application of the ritonavir-boosted nirmatrelvir may be aided by the already-existing guidelines and the clinicians’ experiences in the treatment of HIV-infected patients in order to decrease the risks (e.g., side-effects or drug–drug interactions) [116].

## Figures and Tables

**Table 2:** Sequence variations of the SARS-CoV-2 Mpro cleavage sites of the polyprotein. The P5-P5’ cleavage site residues are shown, the circulating variants are indicated, and the variant counts are shown in parentheses based on COVID-3D resource. n.a., data are not available; -, no variant at this position is included in the database.

	P5	P4	P3	P2	P1	P1’	P2’	P3’	P4’	P5’
nsp4-nsp5	S	A	V	L	Q	S	G	F	R	K
	S496L (3)	A497S (1)	V498I (25)	L499A (1)	-	-	-	F3L (1)	R4K (11)	K5E (1)
			V498F (1)						R4G (1)	
nsp5-nsp6	G	V	T	F	Q	S	A	V	K	R
	G302C (8)	V303I (4)	T304I (54)	F305L (1)	Q306H (4)	S1N (127)	-	V3M (25)	K4E (36)	R5G (13)
	G302S (1)	V303G (1)	T304N (35)	F305C (1)	Q306R (1)			V3L (10)	K4R (21)	
			T304P (1)					V3I (5)	K4Q (6)	
								V3A (4)	K4G (1)	
nsp6-nsp7	V	A	T	V	Q	S	K	M	S	D
	V286I (22)	A287T (35)	T288I (22)	V289L (20)	Q290H (90)	-	K2R (4)	M3I (1096)	S4A (7)	D5A (7)
	V286L (8)	A287V (32)	T288A (3)	V289I (7)			K2E (2)	M3T (12)	S4L (3)	D5E (4)
		A287S (2)	T288S (3)	V289E (1)				M3V (3)		
		A287D (1)	T288N (2)							
nsp7-nsp8	R	A	T	L	Q	A	I	A	S	E
	R79M (5)	A80V (250)	T81I (257)	L82S (2)	Q83H (3)	-	I2V (20)	A3V (26)	S4L (37)	E5D (8)
	R79G (4)	A80T (8)	T81A (4)				I2M (6)	A3S (3)	S4T (5)	
	R79S (1)						I2T (3)	A3T (2)	S4A (1)	
	R79K (1)						I2L (2)	A3D (1)		
nsp8-nsp9	A	V	K	L	Q	N	N	E	L	S
	A194V (74)	V195I (35)	-	-	Q198H (31)	-	-	-	-	S5G (11)
		V195F (3)								S5N (5)
		V195A (1)								S5R (1)
nsp9-nsp10	T	V	R	L	Q	A	G	N	A	T
	T109I (287)	V110L (1)	R111H (1)	L112T (1)	Q113R (1)	n.a.	n.a.	n.a.	n.a.	n.a.
	T109R (1)	V110N (1)	R111C (1)							
nsp10-nsp11	E	P	M	L	Q	S	A	D	A	Q
	n.a.	n.a.	n.a.	n.a.	n.a.	n.a.	A2V (44)	D3G (12)	A4V (54)	Q5P (1)
							A2T (5)	D3N (6)	A4T (4)	Q5K (1)
							A2S (5)	D3E (1)	A4S (1)	Q5L (1)
							A2D (2)		A4E (1)	Q5R (1)
nsp11-nsp12	H	T	V	L	Q	A	V	G	A	C
	H928Y (100)	T929I (2)	V930I (14)	L931T (1)	Q932H (41)	-	V2I (3)	G3R (1)	A4V (152)	C5S (1)
	H928Q (4)	T929A (1)	V930F (1)	L931Y (1)					A4S (41)	
	H928L (2)	T929S (1)	V930L (1)						A4T (1)	
nsp12-nsp13	V	A	T	L	Q	A	E	N	V	T
	V597L (13)	A598S (6763)	T599I (744)	L600Y (1)	Q601H (2)	-	E2A (1)	N3T (6)	V4L (5)	T5I (9)
	V597A (11)	A598V (418)	T599N (10)		Q601K (1)		E2G (1)	N3S (2)	V4I (4)	T5A (6)
	V597M (3)	A598T (10)	T599A (1)				E2K (1)	N3H (1)	V4A (1)	
	V597W (1)	A598E (1)	T599L (1)				E2Q (1)	N3I (1)		
		A598L (1)					E2V (1)			
		A598Q (1)								
nsp13-nsp14	F	T	R	L	Q	S	L	E	N	V
	F523C (1)	T524I (114)	R525K (32)	L526F (2)	Q527P (1)	-	-	-	-	-
	F523L (1)	T524A (2)	R525I (3)	L526P (1)						
		T524Q (1)	R525D (1)							

## Data Availability

Not applicable.
